# 

**Published:** 1813-04

**Authors:** 


### MONTHLY CATALOGUE OF MEDICAL BOOKS.

A TREATISE on the Diseases and Organic Lesions of the Heart and Great Vessels. By J. N. Corvissart, M.D. &c. &c. Translated from the French by J. H. Hebb, Member of the Royal College of Surgeons in London. 8vo.—Underwood.

An Appendix to an Enquiry into the present State of Medical Surgery. By the late Thomas Kirkland, M.D. In which the removal of obstructions and inflammation in particular instances, with the causes, nature, distinction, and cure, of Ulcers, are considered. Taken from his Manuscripts, with a preface, introduction, notes, &c.; by James Kirkland, Surgeon-Apothecary to the Tower. 8vo.—Underwood.

A Treatise on the Management of Female Complaints. By Alexander Hamilton, M.D. Professor of Midwifery in the University of Edinburgh, &c. &c. &c.; seventh edition, revised and enlarged: with Hints for the Treatment of the principal Diseases of Infants and Children. By Dr. James Hamilton, jun. Professor of Midwifery in the University of Edinburgh, &c. 8vo.—Underwood.

Hints for the Treatment of the principal Diseases of Infancy and Childhood, adapted to the Use of Parents. By James Hamilton, M.D. Professor of Midwifery in the University of Edinburgh. 8vo.—Underwood.

Synopsis Nosologiæ Methodicæ exhibens Clariss. Virorum Sauvagesii, Linnæi, Vogelii, Sagari, Macbridii et Culleni, Systemata Nosologica, ordine systematico planius quam antehac disposita. Auctore Gulielmo Cullen, M.D. et nuper in Acad. Edin. Med. Pract. et Medico Regio apud scotos Primario, &c. &c. Editio Nova, cui tabula synoptica systematis culleniani adjicitur. 8vo.—Underwood.

Pharmacopeia Collegii Regii Medicorum Edinburgensis. 18mo.—Underwood.

An Examination of the Imposture of Anne Moore, called the Fasting Woman of Tutbury; illustrated by Remarks on other Cases of real and pretended Abstinence. By Alexander Henderson, M.D. Physician to the Westminster General Dispensary. 8vo.—Underwood.

A Treatise on Febrile Diseases, including Intermitting, Remitting, and Continued Fevers; Eruptive Fevers; Inflammations; Hemorrhagies; and the Profluvia. In which an attempt is made to present, at one view, whatever, in the present state of Medicine, it is requisite for the Physician to know respecting the symptoms, causes, and cure, of those Diseases. By A. P. Wilson Philips, M.D. F.R.S. Edin. Fellow of the Royal College of Physicians, Edinburgh. A new Edition, improved, in 2 vols. 8vo.—Underwood.

List of a Select Collection of Scarce and Valuable Books in Anatomy, Medicine, Surgery, Midwifery, &c. &c. in various Languages; many of which have been recently imported. To be had Gratis.—Underwood.

Outlines of the Anatomy of the Human Body, in its Sound and Diseased State. By Alexander Monro, jun. 3 vols.—Constable and Co.

Elements of Chystallography, after the Method of Haüy; with or without series of Geometrical Models, both Solid and Dissected. By Frederick Accum, Operative Chemist. With Copper-plates. 8vo.—Longman and Co.

A Letter to Sir Francis Milman, bart. M.D. on the Subject of the Proposed Reform in the Condition of the Apothecary and Surgeon-Apothecary, with an Appendix containing the Correspondence between the General Committee and the three Corporate Medical Bodies. By One of the Committee. 8vo.—Callow.

A Letter addressed to Charles Short, esq. Chairman of a Meeting of Medical Gentlemen of the County of Bedford, held at the George Inn, Bedford, on Friday the 12th of March, 1813; containing Observations on the Proposed Bill for Regulating the Practice of Apothecaries, Surgeon-Apothecaries, and Practitioners in Midwifery. 8vo.—Callow.

Hints for the Recovery and Preservation of Health. 12mo. —Callow.

A Practical Treatise on Cataract. By John Stevenson. Large 8vo. —Highley and Son.

The London Dissector. The fourth Edition.—Highley and Son.

---

### *NOTICES TO CORRESPONDENTS.*

*The Apothecaries and Surgeon-Apothecaries' Act having been withdrawn from Parliament, for the present Session, with an intention to be remodelled; we think it useless now to republish, as we promised, the Abstract, but to wait for the Bill, under its new form, coming into our hands. As several months will now necessarily elapse before it can again be presented, we hope to have the subject extensively elucidated by our Correspondents, before that period arrives. It is confessedly one of much importance, and fully deserving their attention.*

*Communications have reached us from Dr. Pigot; Mr. Fayerman; Studens; &c. &c.*

